# Malaria Knowledge and Treatment Practices in Enugu State, Nigeria: A Qualitative Study

**DOI:** 10.15171/ijhpm.2018.41

**Published:** 2018-05-07

**Authors:** Benjamin Sunday C. Uzochukwu, Edmund Ndudi Ossai, Chinyere Cecilia Okeke, Anne Chigedu Ndu, Obinna E. Onwujekwe

**Affiliations:** ^1^Department of Community Medicine, College of Medicine, University of Nigeria, Enugu Campus, Enugu State, Nigeria.; ^2^Health Policy and Research Group Enugu, Enugu State, Nigeria.; ^3^Department of Community Medicine, College of Health Sciences, Ebonyi State University, Abakaliki, Nigeria.; ^4^Department of Community Medicine, University of Nigeria Teaching Hospital Ituku-Ozalla, Enugu State, Nigeria.

**Keywords:** Knowledge of Malaria, Treatment Practices, Qualitative Study, Enugu State, Nigeria

## Abstract

**Background:** Malaria accounts for 60% of outpatient visits in Nigeria. The aim of the study was to assess the knowledge of malaria and its treatment practices in Enugu state, Nigeria.

**Methods:** Qualitative data was collected through the use of focus group discussions (FGDs), from six villages three each from urban and rural areas of Enugu state, Nigeria. A total of 18 FGDs involving 189 participants were conducted and data on place of treatment for malaria and drug of choice for malaria treatment were collected.

**Results:** Most discussants had a good knowledge of the signs and symptoms of malaria. They reported late for treatment when they had symptoms suggestive of malaria. Treatment timing was affected by financial capability and perceived severity of disease. There was preference for patent medicine dealers (PMDs) and pharmacies for malaria treatment. The reasons included drug affordability, obtaining preferred drug, short waiting time and polite treatment from the providers. Treatment in most cases was without proper malaria diagnosis. Cost was an important factor in determining the drug of choice for malaria treatment. This could explain why people were not aware of the use of artemisininbased combination therapy while preferring mono-therapies and herbal drugs. Public hospitals were considered as good sources of treatment for malaria although they remain the last resort when treatment from these drug outlets failed.

**Conclusion:** The community members preferred PMDs and pharmacies for malaria treatment. Unfortunately, these drug outlets do not encourage the use of artemisinin combination therapy (ACT). This makes it necessary that pharmacists and PMDs are trained on management of malaria. Also, improving the knowledge of the public on the need for malaria diagnosis before treatment and use of artemisinin-based combination therapy will improve the control of malaria. The populace should be instructed to seek treatment early while also discouraging the use of herbal drugs for malaria treatment. There is also the need to improve service delivery at public health facilities.

## Background

Nigeria bears more than a quarter of the global burden of malaria.^1^ Nationally, 97% of the population are at risk of the disease.^2^ The result is that malaria accounts for an estimated 60% of out-patient hospital visits, 30% of hospital admissions and 11% of maternal mortalities in the country.^3^ Malaria also has a deteriorating effect on the economy as an estimated 132 billion Naira (700 million dollars) is lost due to treatment costs, prevention and other indirect costs.^4^

To reduce the malaria burden, the World Health Organization (WHO) recommends use of artemisinin combination therapy (ACT), for uncomplicated malaria that is confirmed with either microscopy or rapid diagnostic test (RDT), in a bid to reduce the misuse of ACT.^5,6^ The Nigerian government adopted the WHO guidelines on the use of ACTs for the treatment of uncomplicated malaria in 2005.^7^ Unavailability and inaccessibility of healthcare facilities especially in rural areas in Nigeria is a limitation to provision of appropriate and effective malaria treatment services.^8,9^ Malaria treatment is sought through different healthcare providers including government accredited health facilities and drug retail outlets like pharmacies and patent medicine dealers (PMDs).

Studies have established that these drug retail outlets are usually ineffective in the management and treatment of malaria.^10-13^ PMDs refer to licensed retail outlets whose attendants are not trained pharmacists but can sell over the counter drugs such as analgesics and anti-malarials.^14^ Pharmacies are also medicine retail outlets attended by trained pharmacists whose practice is governed by pharmacy law in Nigeria.^15^ Most patients often prefer this informal sector due to their availability, accessibility and lower cost of treatment although treatment may be inappropriate and practice may be inconsistent with national treatment guidelines.^14,16,17^

Increased utilization of the informal sectors in management and treatment of malaria has become a major public health problem. For instance, both seller and consumer may not have requisite knowledge of appropriate dosage and duration of treatment. In-order to improve malaria case management within communities, strategies such as health education to caregivers and training of community volunteers on management of malaria in children have been used.^18,19^ Health education has also been found to positively influence early diagnosis and appropriate treatment in home management of malaria by caregivers.^20^ If understood, the current treatment seeking behaviour could be useful in informing design of strategies and programs on improving provision and utilization of appropriate malaria case management. The purpose of this study was to determine the knowledge of malaria and its treatment practices in urban and rural areas of Enugu state, Nigeria.

## Methods

### Study Area

The study was conducted in Enugu state in the southeast geo-political zone of Nigeria. It is made up of seventeen local government areas, five of which are urban while twelve are rural. (see Figure). Inhabitants were mainly of Igbo ethnic nationality and predominantly profess the Christian faith. Their main livelihood activities were farming, fishing and wine tapping. Enugu state operates the district health system which is an administrative and management arrangement that merges primary and secondary healthcare. Healthcare services are mostly provided through primary health centres under the authority of the local government councils, secondary health facilities which are controlled by the government of Enugu state and tertiary hospitals which are owned by the federal government of Nigeria. There are also private health facilities, PMDs and pharmacies. Major sources of malaria treatment in the state include public and private health facilities, pharmacies and PMDs. The pharmacies and PMDs do not offer diagnostic or clinical care but are basically medicine retailers although they do recommend drugs to clients. Enugu state has a high malaria transmission rate all year round.

**Figure:**
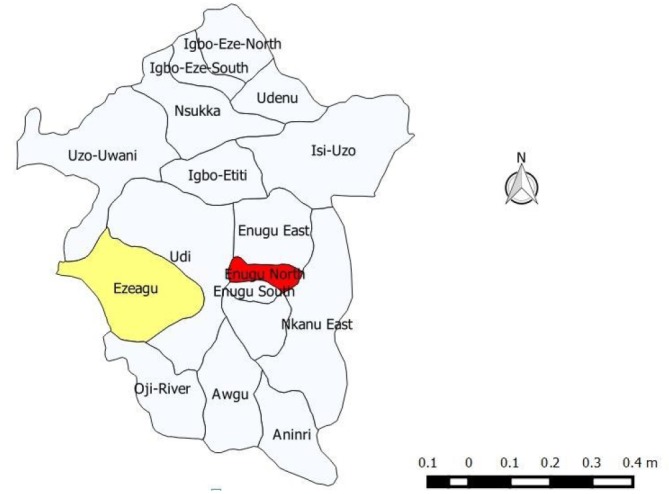
Map of Enugu State and Study Site.

### Sample Selection

A multi-stage sampling method was used in the study. In the first stage, a simple random sampling technique was used to select one local government area from the urban and rural areas of the state. In the second stage, three villages were selected from each of the selected local government areas using simple random sampling technique. Participants were then purposively selected to represent three target groups in each of the villages selected for the study. All participants were aged 25 years or more and were categorized into three groups: women with children under 5 years; women without children; and men. They were drawn from the selected villages by contact persons who were carefully identified by the research team.

### Study Design

This was a cross-sectional qualitative study. Data was collected through focus group discussions (FGDs) with the three categories of discussants and the help of an FGD guide. A total of 18 FGDs were conducted, six with every discussant category. Of the six, three were with discussants from rural areas and the other half from urban areas. Each FGD had an average of 10 participants.

### Study Participants

The study consisted of 189 participants who resided in the selected urban and rural areas of Enugu state, Nigeria. This included 63 male and 116 female participants. The participants must have lived in the selected villages for more than two years. This was considered adequate for them to have understood how the people in this area managed malaria.

### Data Collection

During the FGDs data on malaria knowledge levels and treatment seeking practices were collected. Before data collection, the FGD guide was pretested in two different villages not selected for the study. This was to ensure that there were no ambiguities in the questions. The FGDs were conducted by the researchers in the local language (Igbo), and were conducted in secluded places like public primary schools and community town halls and lasted between 45 to 60 minutes each. All FGDs were recorded manually and using recorders.

### Data Analysis

The recorded discussions were transcribed verbatim following each session by transcribers and then translated to English by two native speakers with good command of both languages.

For quality assurance purposes, the scripts were compared with the written notes for completeness and accuracy. Further, each script was checked against the audiotape by an independent reviewer. In-order to verify the quality of translations, tapes were doubly transcribed after which both scripts were checked for similarity and where differences existed, these were reconciled by the transcribers. Coding of transcripts was done based on emergent themes. Five themes emerged from the study and included malaria signs and symptoms in adults and children, when to seek treatment for adults and children and where to seek treatment for adults and children. Others included communities’ use of PMDs and pharmacies for treatment and medicines used for malaria treatment.

### Limitations of the Study

Use of FGD only could have affected the results of the study. For example, the non-random selection of discussants means that the study findings cannot be generalized to the entire Enugu state population. The researchers had no control over the participants selected by community contact persons despite having given them a selection criterion and thus there may have been a selection bias. Not eliciting the cause of malaria from the participants is a weakness on its own. Furthermore, data was collected only from community members with none from the operators of pharmacies and PMDs.

## Results

### Participants’ Profile

The age range of study participants in the urban area was 26 to 42 years and a median age of 33. In rural areas, participants’ age range was 28-49 years, with a median of 39. The majority of the participants had completed secondary education.

### Malaria Signs and Symptoms in Adults and Children

Most study participants identified headache and fever as symptoms of malaria.

Other symptoms correctly recognized by the participants included weakness, cold, body pains and dizziness as signs and symptoms of malaria. In fact, a participant particularly paid attention on fever as the main symptom of malaria. This was expressed this way:

“*I have found that not all fevers respond to paracetamol (an analgesic), so anytime I have fever, the first thing I do is to take paracetamol, if it does not go then I will conclude that it is malaria and take the necessary actions”* (Female discussant, rural area).

A caregiver reported that decreased appetite is a characteristic of malaria as follows:

“*Anytime my child is not eating well as expected my first reaction is to suspect malaria, I will first give the child paracetamol, (an analgesic) after which I will watch the child closely. If the child starts eating well then the malaria is gone but if the child tries to vomit or vomits or is not sleeping very well then I will conclude it is malaria and I will take the child to the health centre”* (Female caregiver, urban).

Some signs and symptoms assumed to be malaria by the respondents are actually not. These include having a bitter taste in the mouth, urine being yellow in colour in adults and catarrh in children which is interpreted as headache hence a symptom of malaria as exemplified by the following quotes:

“*For me whenever my mouth is bitter, it is a sign that I am about to have malaria and if there is a feeling that I am about to vomit or that my stomach is not alright then it is clear to me that I have malaria”* (Female discussant, urban area).

“*When my child or any other child is crying too much and especially when this cannot be controlled, it means the child is restless hence unable to eat well and whether there is fever or not, the child has malaria”* (Female discussant, rural area).

Two of the participants were of the opinion that people react differently to malaria meaning that symptoms vary depending on each individual. One of the participants had this to say:

“*Different bodies have different ways of reacting to sickness like malaria, some would manifest with headache, if you touch the baby, they will be very hot. And you would like to know if malaria has been treated”* (Female discussant, rural area).

### When to Seek Treatment for Adults and Children

Study participants reported that they sought malaria treatment at different times. Some noted they seek treatment at the onset of malaria symptoms but most would wait for a day or two. A participant described her own action this way:

“*Once I find myself having these symptoms that I have mentioned, I will rush to the nearest ‘ore ogwu’ (meaning a seller of medicine, PMD) and will ask him to give me Camoquine”* (Female discussant, urban area).

Few discussants admitted going to either hospital or health centre for treatment a day or two after the onset of malaria symptoms as exemplified by the following three quotes:

“*Anytime I perceive malaria symptoms, I usually wait for one or two days as a way to confirm that it is malaria. If feverish feeling continues then I will go to a private hospital to be properly taken care of by the doctors” (Male discussant, urban area).*

“*If I have fever or headache, I will take drugs like paracetamol, (an analgesic). However, if these symptoms do not go away but increase I will know that it is malaria and I will go for treatment*” (Female discussant, urban area).

“*When I feel tired, I usually take panadol, (an analgesic), and sometimes the tiredness will go (and that means it is small malaria) but if it persists to the extent of causing generalized body weakness it means it is a strong malaria and that means I will go for treatment”* (Female discussant, rural area).

Some of the participants made it known that their ability to seek treatment depended on financial capability and this affects the health seeking behaviour. This was captured this way:

“*For me, it depends on my financial condition at that particular time, if I have money I commence treatment immediately, but if I don’t have money, I try to withstand the sickness, then it is only when it becomes too much that I will have no other option but to seek for treatment”* (Male discussant, rural area).

### Where to Seek Treatment for Adults and Children

Most of the participants visit health centres, public hospitals, pharmacies and PMDs when they or their children are sick with malaria. One of the participants expressed it this way:

“*I usually go to the health centre for treatment. It is the same place where I take my children for immunization and for treatment when they are sick. You know the nurses there have a good way of taking care of us”* (Female discussant, urban area).

A caregiver admitted home management of malaria based on a previous prescription from a doctor. This was how she presented it:

“*When my child was small, I took him to hospital because he had malaria, the doctor sent us for a laboratory test. After the test, the doctor prescribed chloroquine. So since then, I’ve been giving my child chloroquine whenever he falls sick and it usually stops the malaria”* (Female discussant, rural area).

Two participants in urban area reported undergoing testing and treatment in health facilities. However, health providers determined the time of commencement of treatment. This was presented this way:

“*I go to the health centre with my child and what the nurse does is to send us to the laboratory. If the malaria is serious she will start treatment as we wait for the result. If the malaria is not serious we come with the test result the next day for treatment”* (Female discussant, urban area).

Another two participants also from the urban area preferred the services of private health providers in malaria treatment. The preference was based on short waiting time and use of injections for treatment. This was expressed this way:

“*Whenever my child has malaria, I go to a private hospital for treatment and this is because they attend to you faster there unlike the government hospitals and most times they use injections which work well and faster than tablets” (Female discussant, urban area).*

A large number of discussants identified PMDs as the usual place for malaria treatment. A participant admitted in her words:

“*I prefer to go to medicine vendors. They have a way of combining medicines and with small money you buy a lot of medicines that will help you get well. They only ask a few questions”* (Female discussant, rural area).

Some primary caregivers especially in the rural area also admitted that PMDs are the first source of malaria treatment as demonstrated by the following quotes:

“*We go to one man called Eugene to mix drugs for the child, but after 3 or 4 days if it persists, then we take the child/children to the hospital for laboratory test or any other treatment”* (Female discussant, rural area).

“*We do our best to prevent malaria before it comes, but if we mistakenly get malaria, we go to* ‘ore ogwu’* (meaning a seller of medicine, PMD) or midwife near here, and they give us drugs, then after 3 days, if it does not stop, we go to hospital to see the doctor that he might send us for a laboratory test”* (Female discussant, rural area).

Few discussants noted they would undergo a malaria diagnosis and obtain a prescription from the laboratory technician or simply indulge in self-medication.

“*I prefer to go to a laboratory whenever I am sick including malaria and this is because they usually run some tests from where they will know the correct type of medicine to use to treat the sickness”* (Male discussant, urban area).

Some of the participants admitted another form of home management of malaria, this time the use of herbal preparations for the treatment of malaria. This was presented this way:

“*Since I know I will most likely have malaria every two or three months, I usually buy my herbal preparations and have them ready, however if the malaria comes suddenly, I will prepare the ‘agbo’ (herbal preparation) myself”* (Male discussant, rural area).

The place to seek for treatment is also related to the financial condition of the individual which is determined by the cost of treatment. According to a participant:

“*It depends on the amount of money you have. If you don’t have money you go to the PMD and buy medicine even if it is for 50.00 Naira (0.25 USD). But if you have money, you go to the hospital to see the doctor or you can just go to the health centre because they don’t usually charge money at the health centre”* (Female discussant, rural area).

Differences also existed between the health seeking behaviours of urban and rural dwellers from the results of this study. Use of formal health facilities for malaria treatment and the idea of investigating the cause of fever before treatment were recorded more for inhabitants of urban areas. People in rural areas mostly preferred PMDs and also relied more on herbal mixtures for malaria treatment.

### Communities’ Use of Patent Medicine Dealers and Pharmacies for Treatment

The major reason given by discussants for use of PMDs for malaria treatment was ability to purchase affordable drugs. Other reasons included ability to purchase a preferred drug, short waiting time before treatment and considerate and polite treatment by PMDs. Treatment costs favour use of PMDs as made known by a discussant:

“*Some go to ‘ore ogwu’ (meaning a seller of medicine, PMD) it depends on the amount of money you have. If you don’t have money you go to the medicine vendor and buy drugs even if it is 50.00 Naira (0.25 US dollars) drug”* (Female discussant, rural area).

Also, the PMDs seem to have adapted themselves to the community thus making them a delight to the people. One of the discussants captured it this way:

“*The chemist shops are open for longer duration and they are like one of us, we can even wake the attendant up at odd hours if there is the need and we can pay them for the drugs we purchased over a period of time”* (Male discussant, rural area).

Some participants argued that PMD outlets are nearer to their different homes and this has enhanced their patronage as outlets for malaria treatment. A participant reported that:

“*I go to* ‘*ore ogwu’ (meaning a seller of medicine, PMD) because the hospital is not near to us here. Parklane and UNTH (tertiary hospitals in Enugu) are far so we patronize surrounding medicine shops”* (Male discussant, urban area).

Some primary caregivers noted difficulty at accessing treatment at public health facilities as a main reason for using PMDs for malaria treatment, one of the caregivers expressed her frustration on the treatment given in public hospitals this way,

“*At government hospitals, sometimes they will see that you are with a sick child and doctor is inside, he will look at his watch and if *it* is around 3:30*pm*, he will leave. If you come the next day, they will start from where they stopped. You will go to the hospital for 3 days without seeing the doctor. When you get back home, you pray to God and go and buy drugs from* ‘*ore ogwu’ (meaning a seller of medicine, PMD)”* (Female discussant, urban area).

Most discussants agreed they relied on pharmacies for malaria treatment because they trust the drugs they purchased. Two participants shared their experiences:

“*My experience is that ‘ore ogwu’ (meaning PMDs), and pharmacies are not the same so I go to pharmacy who will give you good drugs”* (Male discussant, urban area).

“*What the pharmacist will do is to look for another drug and combine it with the one I want to buy. I think it works for me that way”* (Male discussant, urban area).

### Medicines Used for Malaria Treatment

The drugs used by the community members for the treatment of malaria included artesunate, chloroquine and brands of sulphadoxine-pyremethamine like malareich, fansidar and maloxine. Surprisingly, no participant indicated taking the recommended anti malaria, ACT for malaria treatment. The choice of medicine for the treatment of malaria by the community members was influenced by several factors. Cost is a determining factor in the choice of anti-malarial drug and this in some instances is also related to the availability of the drug. One of the participants remarked:

“*I also prefer amalar, (a brand of* sulphadoxine-pyremethamine) *it is affordable, always available in the stores and there is nothing like not remembering to take your medicine”* (Female discussant, urban area).

The perceived efficacy of the drug by the participants also encourages them to make use of certain medicines for the treatment of malaria. According to one participant:

“*The best treatment I know, I buy paracetamol (analgesic) and Amalar (a brand of sulphadoxine-pyremethamine)*,* and they cure me of malaria”* (Male discussant, urban area).

This perceived drug efficacy is also the reason why some of the participants prefer the use of herbal preparations for the treatment of malaria. A participant shared her experience this way:

“*This malaria is better cured using herbs, and these herbs have the capacity of going round the body and cure other diseases like typhoid or hepatitis all of which will be expensive to treat if you go to a hospital”* (Female discussant, rural area).

From the views of the participants, the use of herbal drugs has several advantages including absence of adverse effects and affordability. One of the participants expressed it this way:

“*Unlike these chemicals that are called drugs, herbal mixtures are natural hence they have no side effects, they are also cheaper and lasts over a long period of time and it could be taken several times and by this, the malaria is always cleared”* (Male discussant, urban area).

The fear of side effects also limit the use of some of the drugs by the participants and this was pointed out this way:

“*But just as my brother said concerning chloroquine, I for instance don’t take it due to its reactions to me, consumption of any medicine containing chloroquine causes me to itch for four days. I take amalar (a brand of sulphadoxine-pyremethamine) for my malaria treatment which is good for me” (Male discussant, urban area).*

A participant who patronises PMDs prefers the use of injections based on his perceived severity of malaria. From his comments, it could be deduced that he indicates his choice of medicines. He said:

*“*… *for me, I go straight to* ‘ore ogwu’* (meaning PMD) and demand for chloroquine injection which works very well for me or fansidar (a brand of* sulphadoxine-pyremethamine) *if I think that it is not a serious malaria”* (Male discussant, urban area).

Another male participant in the rural area supported this view by stating that generally injections work better and faster than tablets any time.

A few of the participants relied more on prevention of malaria rather than its cure. Unfortunately, their understanding of preventive measures is questionable. One of the participants presented it this way:

“*The best treatment is for everybody to be careful with the type of food and water that we take but the major one is the water that we drink and the environment where the person is living. All these could contribute to our being sick with malaria” (Female discussant, urban area).*

Some of the participants could not remember the names of the drugs they usually take for malaria treatment. These may be the people who present to the PMDs or pharmacies and rely on their expertise for treatment. They may also include those who present at private health facilities for treatment as they may make do with the doctor or any other health practitioner’s choice of drugs since they perceive that they may be guided by the result of a laboratory test.

## Discussion

From the results of this study, headache and fever were identified as the major symptoms of malaria. This is similar to the results of other studies in which fever was regarded as one of the main symptoms of malaria.^21,22^ Even though the community members had good knowledge of malaria symptoms, certain misconceptions still exist like the reliance on yellowish urine, bitterness of mouth which are very subjective as symptoms of malaria in adults and in children, the linking of catarrh to headache hence interpreted as a symptom of malaria. Elsewhere it has been found that the knowledge of severe symptoms of malaria among the participants was very poor,^21^ and unlike the study among undergraduates in Nigeria,^21^ no participant in this study mentioned coma, convulsion and anaemia which are complications of malaria as symptoms of malaria.

Similarly, the comment on prevention of malaria in which the disease was linked with air and water is also misleading. It has been found that misconceptions on cause and transmission of malaria still exist among the people,^23^ and this necessitates that studies of this nature should begin with eliciting the perceived cause of malaria from the participants. Also based on the observation that knowledge of cause of malaria affect health seeking behaviour, there has been a call for the enlightenment of the people on the transmission of malaria as this will influence health seeking behaviour.^24^ Interestingly, a study in Malawi also identified the need to improve the knowledge of the symptoms of malaria among caregivers.^25^ As such emphasizing the need to intensify malaria enlightenment programmes.^26^

Most community members did not receive adequate malaria diagnosis. It has been found that in Africa most patients with fever, which could be suggestive of malaria, present at the informal sector where malaria diagnosis using RDT is not very common.^27,28^ However based on the acceptance of RDT by the drug outlets and members of the community, there is need to educate the public on the use of RDT at the community so as to improve malaria case detection.^28^ Thus use of RDT in drug retail outlets has been advocated for,^27^ and this is in line with one of the pillars of the WHO global strategy for malaria which is to ensure universal access to malaria prevention, diagnosis and treatment.^29^ This approach includes the scaling up of community based diagnostic testing and treatment of malaria,^29^ which could be achieved with the involvement of the drug retail outlets in management of malaria.

The delay of two or three days in seeking for treatment for symptoms suggestive of malaria among the participants may be attributed to the effect of a popular advertorial for a well-known analgesic which was repeatedly aired on the electronic media and the message requested the populace to consult a physician after three days if symptoms persisted after the use of the analgesic. It is important to note that the advertisement was not for malaria treatment but may have been misunderstood by the people. At that period also, there was no emphasis on malaria diagnosis using RDT or microscopy before treatment. Since the people are consciously holding on to the values of that advice, there may be the need for increased public awareness on the current World Health Organization guidelines on the diagnosis and treatment of malaria. This is important in changing the perception of the people on use of analgesics thus encouraging the members of the public to make right choices for malaria treatment. This has necessitated the call for interventions that will improve the awareness of caregivers on the relevance of seeking medical care early and advocating for early diagnosis and treatment for malaria. This obviously will reduce the likelihood of complications of malaria.^30^

Another hindrance to seeking treatment early by the participants may be related to cost. In Nigeria, out of pocket expenditure for health is the main source of financing for health and this is irrespective of the fact that majority of the populace live in abject poverty.^31^ Undoubtedly, this has been identified as a barrier to accessing healthcare,^32^ hence in a study that involved government employees in southeast Nigeria, who are more enlightened than the average population, those who relied on out of pocket expenditure had difficulties assessing quality healthcare services.^33^ Perhaps, the introduction of alternative health financing mechanisms will help in ensuring universal access to malaria prevention, diagnosis and treatment and to healthcare services generally. This has already been recognised as a barrier to the utilization of formal heath care system.^34^

PMDs and pharmacies were mostly used by community members for malaria treatment. These two drug retail outlets have already been identified as the main service providers for the treatment of malaria.^35^ Unfortunately, some of these providers stock several anti-malarial drugs including those that are not recommended for treatment.^36^ The result is their reliance on mono-therapies for the treatment of malaria. Several factors have been known to influence the choice of anti-malarial drugs by the PMDs and most important one is the personal choice of the vendor.^37^ Other factors included influence by patients, representatives of drug companies and other providers working at the same facility or area.^38^ The stocking of anti-malarial drugs by the providers may also have been influenced by cost as the retail drug outlets being privately owned may be concerned more on profit than national treatment guidelines.

It has been established that ACTs are more expensive when compared with the mono-therapies.^35^ This may influence the choice of anti-malarial use by the people in favour of mono-therapies. This has already being identified as a challenge in the fight against malaria.^29^ Even among the retail drug outlets, some differences exist. Based on Nigeria law, pharmacies should have the services of a trained pharmacist whose understanding of medicines and of health generally should be considerably higher than that of PMDs. Thus it has been found that poorer individuals seeking care at PMDs are more likely to receive inappropriate treatment when compared with those who go to pharmacies.^39^ The reason why the pharmacies failed to dispense ACTs to clients remain unclear. With this evidence at hand, training of retail drug outlet operators on management of malaria is essential while also creating awareness among the populace on the new malaria treatment policy among the people. In effect, promoting affordable treatment for malaria is of prime importance,^26^ and the drugs should be available always.^8^ Hence there has been a call for the reduction of the cost of effective drugs for the treatment of malaria, in this case ACTs, and increasing its acceptability among the populace.^40^

When compared with the PMDs and pharmacies, the public health facilities offer the highest quality of malaria treatment services to the people.^35^ Based on this position, families are more satisfied with malaria treatment services from the public health facilities. The provision of quality assured treatment for all malaria patients has been identified as one of the strategies for the control of malaria.^29^ This may explain why public health facilities are places of last resort when it becomes obvious that malaria treatment services from these retail drug outlets have failed or are about to fail. This not-withstanding, the experiences of the participants in these facilities have not been good. This has made it necessary to insist on the delivery of quality health services from public health facilities in Nigeria. Already there have been calls for the strengthening of the health systems in Nigeria,^34^ as a way of ensuring quality health service delivery.^8^

People in urban and rural areas seek for treatment of malaria in different treatment outlets with the rural dwellers preferring PMDs.^8,41^ The rural dwellers also prefer cheaper drugs,^41^ and this may inform their reliance on herbal drugs for malaria treatment. These differences may be due to the gap between the people living in urban and rural areas of Nigeria when educational attainment and socio-economic status are considered.^2^ For example in a study in a rural area in Nigeria a higher proportion of the respondents used herbal drugs to treat malaria when compared with those who used modern medicines.^42^

## Conclusion

Community members preferred PMDs and pharmacies for malaria treatment. Unfortunately, these drug outlets do not encourage the use of ACT. This makes it necessary that pharmacists and PMDs are trained on management of malaria. Improving public knowledge on the need for malaria diagnosis before treatment and use of artemisinin-based combination therapy for treatment will improve the control of malaria. The populace should be instructed to seek treatment early while also discouraging the use of herbal drugs for malaria treatment. There is also the need to improve service delivery at public health facilities and introduce alternative financing mechanisms for healthcare thus reducing out of pocket expenditure for health by the people.

## Ethical issues

Ethical approval for the study was obtained from the Health Research and Ethics Committee of University of Nigeria Teaching Hospital Ituku-Ozalla, Enugu with approval number NHREC/05/01/2008B-FWA0002458-IRB00002323. Participation in the study was voluntary. All the participants were above the consenting age of 18 years. Prior to signing an informed consent form, participants were informed of the nature of the study, its relevance and the methods that would be used in data collection. In addition, they were assured that the data collected during the discussions would be confidential and that there would be no victimization of any kind for those who refused to participate or those who decided to withdraw from the study after giving consent.

## Competing interests

Authors declare that they have no competing interests.

## Authors’ contributions

BSCU and OEO conceptualized and designed the study. CCO, ACN, and ENO were involved in data collection. BSCU, OEO, and ENO did the statistical analysis while BSCU, CCO, and ACN wrote the initial draft of the manuscript. All authors read and approved the manuscript for submission.

## Authors’ affiliations

^1^Department of Community Medicine, College of Medicine, University of Nigeria, Enugu Campus, Enugu State, Nigeria. ^2^Health Policy and Research Group Enugu, Enugu State, Nigeria. ^3^Department of Community Medicine, College of Health Sciences, Ebonyi State University, Abakaliki, Nigeria. ^4^Department of Community Medicine, University of Nigeria Teaching Hospital Ituku-Ozalla, Enugu State, Nigeria.

## Key messages

**Implications for policy makers**

- The government and its health agencies should ensure an improvement in delivery of public healthcare services through evidence based health policy formulations and training of health workers.
- On malaria management, emphasis should be placed on proper diagnosis of malaria and treatment using artemisinin-based combination therapy.
- Training of pharmacists and patent medicine dealers on malaria treatment so as to improve quality.
- Increase public awareness on management of malaria.

**Implications for the public**

Community members have preference for patent medicine dealers (PMDs) and pharmacies for malaria treatment. Unfortunately, treatment prescribed in these outlets did not include the use of artemisinin-based combination therapy. This is irrespective of the fact that the Nigerian government adopted the World Health Organization (WHO) guidelines on artemisinin combination therapy (ACT) use for treatment of uncomplicated malaria in 2005. This policy however did not forbid the sale of mono-therapies in the country. There is thus a need to improve peoples’ knowledge on the right actions to take when they have signs and symptoms suggestive of malaria and this will go a long way in improving the control of malaria. Efforts should be made to discourage the public from self-treatment of malaria.
